# Hydrogen migration at restructuring palladium–silver oxide boundaries dramatically enhances reduction rate of silver oxide

**DOI:** 10.1038/s41467-020-15536-x

**Published:** 2020-04-15

**Authors:** Christopher R. O’Connor, Matthijs A. van Spronsen, Tobias Egle, Fang Xu, Heath R. Kersell, Judit Oliver-Meseguer, Mustafa Karatok, Miquel Salmeron, Robert J. Madix, Cynthia M. Friend

**Affiliations:** 1 Department of Chemistry and Chemical Biology, Harvard University, Cambridge, MA 02138 USA; 2 Materials Sciences Division, Lawrence Berkeley National Laboratory, 1 Cyclotron Road, Berkeley, CA 94720 USA; 3 School of Engineering and Applied Sciences, Harvard University, Cambridge, MA 02138 USA; 4 Department of Materials Science and Engineering, University of California, Berkeley, CA 94720 USA

**Keywords:** Heterogeneous catalysis, Surface chemistry, Scanning probe microscopy, Materials for energy and catalysis

## Abstract

Heterogeneous catalysts are complex materials with multiple interfaces. A critical proposition in exploiting bifunctionality in alloy catalysts is to achieve surface migration across interfaces separating functionally dissimilar regions. Herein, we demonstrate the enhancement of more than 10^4^ in the rate of molecular hydrogen reduction of a silver surface oxide in the presence of palladium oxide compared to pure silver oxide resulting from the transfer of atomic hydrogen from palladium oxide islands onto the surrounding surface formed from oxidation of a palladium–silver alloy. The palladium–silver interface also dynamically restructures during reduction, resulting in silver–palladium intermixing. This study clearly demonstrates the migration of reaction intermediates and catalyst material across surface interfacial boundaries in alloys with a significant effect on surface reactivity, having broad implications for the catalytic function of bimetallic materials.

## Abstract (web summary)

Species migration across interfacial boundaries can affect the function of bimetallic catalysts. Here the authors report that palladium oxide drives the reduction of silver oxide by facilitating molecular hydrogen dissociation and migration of hydrogen atoms across the Pd–Ag interface with concurrent surface restructuring.

## Introduction

Heterogeneous catalysts are complex materials that generally contain multiple interfaces which can modify the kinetics and selectivity for key reactions, especially for the production and use of molecular hydrogen for energy generation and chemical synthesis^1–6^. The increasing interest in alloy catalysts creates new challenges in understanding the effect of interfaces. Although interfaces between metal nanoparticles and the metal oxide support have been extensively investigated, less is known about interfaces that form on alloys themselves, independent of the support. Phase segregation on metallic alloy nanoparticles can form metal/metal interfaces primarily arising from differences in surface free energy of the metal components and unfavorable mixing enthalpies^7–9^. Under oxidizing conditions, complex metal/oxide and oxide/oxide interfaces can form by phase separation and surface segregation arising from differences in metal–oxygen bonding^10,11^. Therefore, a dynamic understanding of material restructuring and chemical behavior is required to most effectively use the bifunctional character of alloy catalysts.

Prior studies have demonstrated that the interface between metal nanoparticles and the metal oxide support can affect chemical behavior by the creation of specific active sites^12–18^ and direct migration or “spillover”^19^ of reactive species created on one phase to a neighboring phase of differing reactivity^19–25^. The metal/oxide support interface can significantly restructure in reactive gas environments on a short timescale of minutes and long length scales of microns^26^. Catalyst activity can be significantly modified by interface restructuring including changes in coordination environment^27^, catalyst encapsulation^28^, and metal component transport and alloying^29–33^.

Similarly, metal/metal interfaces of alloy nanoparticles can restructure under reactive gas conditions and promote intermediate migration. The interface can significantly reorganize by segregation and dissolution of metal components^34–36^, structural rearrangement^37^, and formation of oxide/oxide interfaces in reactive gas environments^10,11^. The migration of intermediates via hydrogen atom spillover across a bimetallic interface has been demonstrated for several metal/metal interfaces^38–41^. The behavior of bimetallic oxide interfaces in promoting the migration of intermediates, though, may even be more complex than for metal/oxide support or metal/metal interfaces, as we examine in this paper.

Herein, we demonstrate that synergistic effects arising from interfacial energetics dramatically alter reactivity associated with hydrogen atom migration across the palladium oxide/silver oxide interface produced from oxidation of the alloy, resulting in intermixing of metal atoms at the two-dimensional bimetallic oxide interface during the reactive process at room temperature. Specifically, intermixing of palladium and silver plays a critical role in accelerating the rate of reduction of oxidized Ag(111) by molecular hydrogen because of the presence of palladium oxide islands on the silver oxide surface. The increase in reactivity is attributed to the higher activity of palladium oxide for dissociating molecular hydrogen to produce hydrogen atoms, which can either (1) spillover directly onto the silver oxide or (2) react at the palladium oxide boundary with oxygen provided by silver oxide that is supplied to the palladium as palladium oxide is reduced. In parallel, the rate of reduction of palladium oxide is retarded as silver is incorporated into its structure at the interface. The high mobility of silver and silver oxide complexes, even at room temperature, is an important factor in this mechanism^42–44^. The intermixing of palladium and silver that accompanies the acceleration of silver oxide reduction demonstrates the complexity of synergistic effects in catalysis, especially when reducible oxides are involved.

## Results

### Generation of palladium and silver oxide surfaces

**Fig. 1:**
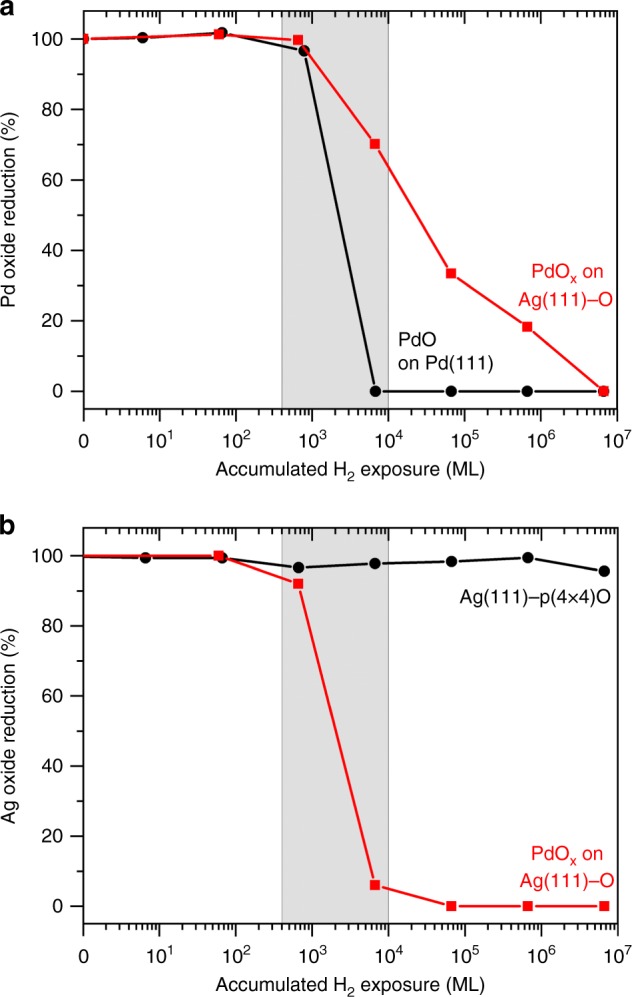
Pd oxide accelerates the reduction of Ag oxide by hydrogen. The degree of reduction of (**a**) palladium oxide in a PdO film on Pd(111) (black) and PdO_*x*_ islands surrounded by Ag(111)-O (red) and (**b**) silver oxide in the Ag(111)-p(4 × 4)O surface oxide (black) and in the silver oxide in the presence of PdO_*x*_ islands with their cumulative exposure to molecular hydrogen at 300 K. The oxidation states of palladium and silver were monitored by the integrated intensities of their respective 3d_5/2_ XPS peaks. The pressure of molecular hydrogen for each reduction step is detailed in the Supplementary Information. All data are normalized to the integrated area of the oxide peaks after the oxidation treatment (Supplementary Figs. 1–4, Supplementary Notes 1–3).

The reactivity of molecular hydrogen with a thin film of palladium oxide (PdO) on Pd(111), silver oxide (Ag(111)-p(4×4)O) on Ag(111) and palladium oxide (PdO_*x*_) on Ag(111) surfaces was compared using ambient-pressure X-ray photoelectron spectroscopy (AP-XPS). A palladium oxide (PdO) film was generated by oxidation of a single crystal of Pd(111) with molecular oxygen at 773 K and 2 Torr (Supplementary Fig. 1)^45^. Similarly, the surface oxide of silver (Ag(111)-p(4×4)O) was generated by oxidation of Ag(111) by molecular oxygen at 773 K and 2 Torr (Supplementary Fig. 2)^46^. Palladium oxide (PdO_*x*_) islands surrounded by the surface oxide of Ag(111) were generated by deposition of 0.10 monolayers (ML) of palladium on Ag(111) followed by exposure to molecular oxygen at 377 K and 3 Torr for 25 min (Fig. 1, Supplementary Figs. 3, 4).

### Palladium dewetting during palladium–silver oxidation

**Fig. 2:**
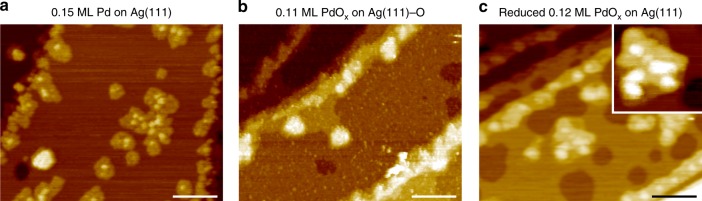
Palladium–silver restructuring during oxidation and reduction. **a**–**c** A series of characteristic STM images of (**a**) as-deposited palladium on Ag(111), (**b**) after oxidation of (**a**) in 3 Torr of molecular oxygen at 425 K, and (**c**) subsequent reduction by molecular hydrogen at 300 K. **a**, **b** The apparent height of the islands increases from single layer (0.2 nm) to multilayer structures (0.9 nm) upon oxidation. **b**, **c** After reduction, the apparent height of the islands decreases to 0.6 nm and etch pits form on the silver attributed to silver oxide decomposition and silver etching and subsequent intermixing into the palladium islands. **c** Inset shows a high-resolution image of the intermixed palladium–silver island. Line scans of the islands and pits are detailed in Supplementary Fig. 6. STM details: (**a**) *V*_sample_ = 2.00 V, *I*_setpoint_ = 0.300 nA, (**b**) *V*_sample_ = 1.50 V, *I*_setpoint_ = 0.300 nA, (**c**) *V*_sample_ = 1.90 V, *I*_setpoint_ = 0.250 nA. Scale bars: 40 nm.

**Fig. 3:**
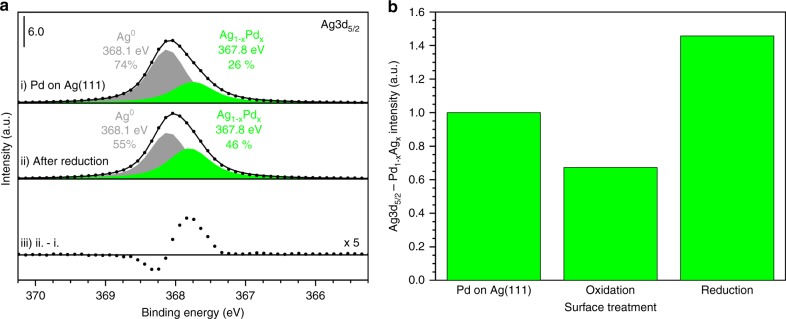
Palladium–silver intermixing during reduction by hydrogen. **a** Palladium–silver intermixing occurs after reduction of PdO_*x*_ on Ag(111) as evidenced by the increased Ag_1−*x*_Pd_*x*_ alloy peak in the Ag3d_5/2_ region (green) using AP-XPS. Spectra are for (i) as-deposited 0.10 ML palladium on Ag(111), (ii) after reduction of PdO_*x*_ on Ag(111), and (iii) the difference trace (ii−i) to better illustrate the increase in the PdAg alloy signal. **b** The formation of PdO_*x*_ on Ag(111) decreases the palladium–silver interface area because of palladium dewetting while the reduction of PdO_*x*_ on Ag(111) induces palladium–silver intermixing as illustrated by the increase in the Ag_1−*x*_Pd_*x*_ alloy peak.

Palladium dewets the surface when the single layer palladium islands on Ag(111) are oxidized to form domains of silver surface oxide and multilayer PdO_*x*_ (Figs. 2a, b, 3). Initially, the palladium forms predominantly single layer islands with an apparent height of 0.2 nm, as revealed by scanning tunneling microscopy (STM) (Fig. 2a). During oxidation, PdO_*x*_ islands form condensed particles with apparent heights of 0.9 nm (Fig. 2b). Accordingly, there is a 30% decrease in the X-ray photoelectron Pd3d_5/2_ total signal relative to palladium on Ag(111) before oxidation, which is attributed to the attenuation of subsurface palladium in the multilayer structures (Supplementary Table 3). The palladium dewetting is associated with a 33% decrease in the Ag3d_5/2_ PdAg alloy signal relative to unoxidized palladium on Ag(111) because of a decreased palladium–silver interface area (Fig. 3b, Supplementary Table 3). Similarly, the segregation of palladium oxide and silver oxide occurs for the oxidation of a Pd_75_Ag_25_(100) bulk alloy^47^.

### Palladium oxide catalyzes the reduction of silver oxide

The key experimental observations that demonstrate hydrogen spillover from islands of PdO_*x*_ onto the surrounding silver oxide surface and the synergistic effects that drive this phenomenon are as follows:

1. Molecular hydrogen is readily activated by palladium oxide. Films of palladium oxide (PdO) on metallic palladium (Pd(111)) were readily reduced by molecular hydrogen, commencing at cumulative molecular hydrogen exposures of approximately 10^3^ monolayers (equivalent) (Fig. 1a, black curve). The reduction of PdO by molecular hydrogen is rapid and autocatalytic, accelerating with time at constant molecular hydrogen pressure^48^. The reaction proceeds through an initial slow rate of molecular hydrogen dissociation on PdO to form hydroxyls and atomic hydrogen, which initiates the reduction of palladium oxide to create a defective surface, which reduces more rapidly based on prior literature^48^.
2. Ag(111)-p(4 × 4)-O is not reduced by molecular hydrogen. Cumulative exposures of up to 10^7^ monolayers of molecular hydrogen cause no observable reduction of surface oxygen (Fig. 1b, black curve), consistent with the low rate of molecular hydrogen dissociation on silver^49,50^.
3. PdO_*x*_ on the Ag(111) surface catalyzes the reduction of the supporting silver oxide by molecular hydrogen. The presence of PdO_*x*_ on Ag(111) increases the rate of reduction of silver oxide with molecular hydrogen by more than 10^4^ at 300 K (Fig. 1b, red curve). Therefore, PdO_*x*_ must activate molecular hydrogen, catalyzing the reduction of the surrounding silver oxide surface at 300 K (Fig. 1a). The onset of reduction of the PdO_*x*_ on Ag(111) occurs at the same molecular hydrogen exposure as does palladium oxide reduction on the PdO surface, which indicates a similar rate for initial molecular hydrogen dissociation on both. PdO_*x*_ is reduced at a much slower rate than occurs on PdO, which will be discussed later in context of structural changes during the reaction (Fig. 1a, red curve). There is clear preference for molecular hydrogen reduction of the surrounding silver oxide rather than reduction of the PdO_*x*_.

### Intermediate migration across palladium–silver interface

The high reactivity of the silver oxide surrounding the PdO_*x*_ islands is attributed to molecular hydrogen dissociation on PdO_*x*_ followed by migration of atomic hydrogen across the palladium–silver interface and reduction of silver oxide. The preferential reduction of the silver oxide surrounding the PdO_*x*_ islands (Fig. 1b, red) occurs at a rate similar to that of the PdO film (Fig. 1a, black), which suggests that the transport and reaction of atomic hydrogen onto the silver oxide must be nearly equal to its rate of generation on the PdO_*x*_. A detailed investigation of the intrinsic kinetics of silver oxide reduction by hydrogen atoms is warranted. Molecular hydrogen reduction of PdO on Pd(111) and palladium oxide^48,51^ occurs via the activation of molecular hydrogen to form atomic hydrogen (and hydroxyls). Hydroxyl migration from PdO_*x*_ to silver oxide and reaction with silver oxide would lead to no net reduction of silver oxide and therefore is implausible. The rate of migration of intermediates across the PdO_*x*_—silver oxide interface is expected to be atomic hydrogen ≈ hydroxyls > atomic oxygen based on their respective binding energies on palladium, silver, and palladium–silver alloy sites^52–61^. However, it is quite possible that atomic oxygen transfers from the Ag(111)-O (perhaps via silver oxide complexes) to the PdO_*x*_ as it is reduced, significantly contributing to the rate of reduction of the silver oxide because it is known that short Ag–O chains are quite mobile on Ag(110), and the reduction of Ag(111)-p(4×4)O by carbon monoxide generates mobile silver atoms^42–44^. Direct hydroxyl migration from silver oxide to PdO_*x*_ to mitigate the rate of reduction of the PdO_*x*_ is unlikely, since it necessitates the formation of hydroxyls on silver oxide by the direct reaction of silver oxide with molecular hydrogen. Therefore, we propose that silver oxide reduction is initiated by atomic hydrogen migration from PdO_*x*_ to silver oxide and that the reduction proceeds by hydrogen atom migration from PdO_*x*_ to silver oxide. The net rate of reduction of the PdO_*x*_ may be mitigated by migration of atomic oxygen from silver oxide to partially reduced PdO_*x*_. The effect of the palladium surface coverage, distribution, and oxidation state on the reduction of silver oxide is currently under investigation.

### Palladium–silver intermixing during reduction by hydrogen

Intermixing of palladium and silver occurs as a consequence of the reduction of the PdO_*x*_ on Ag(111)-O by molecular hydrogen, based on both X-ray photoelectron spectroscopy and scanning tunneling microscopy (Figs. 2b, c, 3). The amount of PdAg alloy relative to palladium deposited on Ag(111) increases after the silver oxide reduction is completed (Fig. 3). Associated with reduction is also the creation of etch pits on the surface. These pits are one-layer deep and cover 38% of the surface (Fig. 2c, Supplementary Fig. 5, Supplementary Table 4, Supplementary Note 4). The combined area of these etch pits greatly exceeds that expected from reduction of the Ag(111)-p(4×4)-O itself, which is known to be only 17% of the surface (Supplementary Table 4)^43^. Thus, the larger fraction of etch pits observed following reduction of palladium oxide/silver oxide is attributed to silver migration to and intermixing into palladium islands during reduction. No measurable changes in morphology of unoxidized, metallic palladium on Ag(111) are observed after exposure to molecular hydrogen at 300 K (Supplementary Fig. 7). Indeed, this intermixing appears to be energetically stable, as it has been observed that heating of palladium islands on Ag(111) is necessary for extensive intermixing^62^. Hence, the intermixing at room temperature must be facilitated by reduction of the palladium and silver oxides (Supplementary Table 4). In support of this intermixing, STM reveals that the islands are significantly higher after reduction of the oxides than they were prior to oxidation (Fig. 2c).

## Discussion

In this work, synergistic effects that dramatically alter reactivity at an oxide/oxide interface of an alloy surface are demonstrated: intermixing of metal atoms at the interface during a reactive process associated with the spillover of a reactive intermediate from one phase to another. The intermixing of palladium and silver occurs during the reduction of oxidized Ag(111) by molecular hydrogen due to the presence of PdO_*x*_ islands.

Previously, migration of hydrogen atoms has been demonstrated away from single metal atoms and metal islands to either a metal or metal oxide host;^19–23,38–41,63^ however, intermixing and reconstruction during reaction was not evident. For example, single palladium atoms in copper promote molecular hydrogen dissociation on palladium and subsequent migration to the copper^38^. Migration of hydrogen atoms also occurs across interfaces between metal particles, e.g. cobalt, and metal surfaces, e.g. copper^39^. Furthermore, migration between metal and metal oxides phases has also been reported for hydrogen atoms^19^. For example, molecular hydrogen dissociation at a site spanning single palladium atoms on iron oxide (Fe_3_O_4_(001)) forms hydroxyls on the oxide via a heterolytic bond breaking process^23^. Migration of intermediates across interfaces between oxide islands of titania and ceria (TiO_2_ and CeO_2_) formed on gold and copper has also been demonstrated^64,65^. Similarly, copper oxidation was promoted by ceria (CeO_2_) islands, indicating that oxygen transport across interfaces is also possible^66^. The intermixing of metal atoms at the interface was not observed in any of these cases.

The acceleration of silver oxide reduction, which is accompanied by intermixing of palladium and silver described here demonstrates the complexity of synergistic effects in alloy nanoparticle catalysis, especially when reducible oxides are involved. Such intermixing can alter reaction rates and, therefore, must be considered in modeling such behavior.

The dramatic enhancement of the rate of silver surface oxide reduction due to the presence of PdO_*x*_ islands is accompanied by intermixing of palladium and silver. The facile intermixing of metals involved during a reaction process on a model alloy surface demonstrates the complexity of synergistic effects in alloy nanoparticle catalysis. Such intermixing, which can alter reaction rates, has not previously been established in the context of reactant migration across the interface between two different reducible oxides formed from oxidation of an alloy. This study clearly demonstrates that such intermixing must be considered in modeling the behavior of complex catalyst materials.

## Methods

### Sample preparation

The Pd(111) single crystal was cleaned by several cycles of argon ion (Ar^+^) sputtering (10–20 min, 1 keV) and annealing (5 min, 1000 K). The Ag(111) single crystal was cleaned by several cycles of argon ion (Ar^+^) sputtering (10–20 min, 2 keV), molecular oxygen treatment (1 × 10^−5^ Torr, 5 min, 500 K) and annealing (5 min, 800 K). Palladium was evaporated and deposited on the Ag(111) crystal when the sample was 300 K. Pd(111) and Ag(111) were oxidized in 2 Torr of molecular oxygen at 773 K for 5 min while palladium on Ag(111) was oxidized in 3 Torr of molecular oxygen at 373 K for 25 min.

### AP-XPS details

AP-XPS experiments were performed at beamline 11.0.2 of the Advanced Light Source at the Lawrence Berkeley National Laboratory. Prior to experiments the chamber was cleaned using a conventional bake-out and between experiments the chamber was repeatedly purged with 2 Torr of molecular oxygen to reduce contamination from the displacement of gases adsorbed to the chamber walls during high-pressure doses. All spectra were recorded in ultra-high vacuum (UHV) in order to eliminate beam-induced reactivity that can occur when a significant gas pressure is present.

A photon energy of 800 eV was used to take a survey scan to check for contamination before acquiring high-resolution spectra. Photon energies of 535 eV for Pd3d and 568 eV for Ag3d were used to produce photoelectrons with kinetic energies around 200 eV for high-resolution spectra. The binding energies reported are referenced to the Fermi level as measured after each spectrum. The data was fitted after a Shirley^67^ background subtraction and normalization to the background at the low binding energy side of the peaks. The Ag(111) and Pd/Ag(111) spectra were fitted with a Doniach-Šunjić function^68^ convoluted with a Gaussian function and the Pd(111) spectra were fitted with a Mahan function^69^ convoluted with a Gaussian function.

### STM details

STM experiments were perform using an Omicron VT-STM-XA 650 system (UHV Multiprobe). All samples were prepared in the preparation chamber of a lab-based ambient-pressure photoelectron spectroscopy system. The samples were then transferred in a vacuum suitcase (base pressure < 1.0 × 10^−9^ Torr) for imaging in the UHV multiprobe surface analysis system.

## Supplementary information

Supplementary Information

Peer Review

## Data Availability

All the data that support the findings of this study are available within the paper and its Supplementary Information, or from the corresponding author on reasonable request.
