# Supplementary material for: Hydrogen migration at restructuring palladium–silver oxide boundaries dramatically enhances reduction rate of silver oxide
Source: Nat Commun. 2020 Apr 15;11:1844. doi: 10.1038/s41467-020-15536-x (PMC7160204; doi:10.1038/s41467-020-15536-x)
Supplement: Supplementary file 1 — Supplementary Information [file 41467_2020_15536_MOESM1_ESM.pdf]

Supplementary Information for
Hydrogen migration at restructuring palladium-silver oxide boundaries dramatically enhances reduction rate of silver oxide

O'Connor et al.

Supplementary Note 1. The facile activation of hydrogen on oxidized Pd(111) leads to the complete reduction of palladium oxide (Supplementary Figure 1). Reduced Pd(111) is characterized by $Pd3d_{5/2}$ peaks at 334.9 ± 0.1 eV and 334.7 ± 0.1 eV, which are ascribed to bulk (Pd^{o}-bulk) and surface palladium (Pd^{o}-surface), and by a $Pd3p_{5/2}$ peak at 531.8 eV ± 0.1 eV, which is attributed to metallic palladium (Supplementary Figure 1).[1] The oxidation of Pd(111) forms a oxygen-terminated palladium oxide surface characterized by a $Pd3d_{5/2}$ peak at 336.2 ± 0.1 eV, attributed to palladium coordinated with four oxygens (Pd-4O), O1s peaks at 531.2 – 530.9 eV, 529.6 ± 0.1 eV and 528.5 ± 0.1 eV 3-fold oxygen, attributed to hydroxyls, 4-fold oxygen and 3-fold oxygen and by a $Pd3p_{5/2}$ peak at 533.2 eV ± 0.1 eV, which is attributed to palladium oxide (Supplementary Figure 1).[2,3] Reduction of this surface was examined by sequential exposures of hydrogen at pressures of 1.1×10^{-7} Torr (6×10^{0} L), 1.0×10^{-6} Torr (6×10^{1} L), 1.0×10^{-5} Torr (6×10^{2} L) and 1.0×10^{-4} Torr (6×10^{3} L) for 1 min at 300 K (Supplementary Figure 1). No reduction occurred until the 6×10^{2} L hydrogen exposure, as evidenced by a slight decrease in the Pd-4O and $O_{3\text{-fold}}$ peaks and the emergence of a $Pd3d_{5/2}$ peak at 335.4 ± 0.1 eV, which can be attributed to palladium coordinated with two oxygens (Pd-2O, Supplementary Figure 1).[2,3] An exposure to 6×10^{3} L of hydrogen induced the complete reduction to metallic palladium, as evidenced by the sole presence of the Pd^{o}-bulk, Pd^{o}-surface and metallic $Pd3p_{5/2}$ peaks (Supplementary Figure 1).

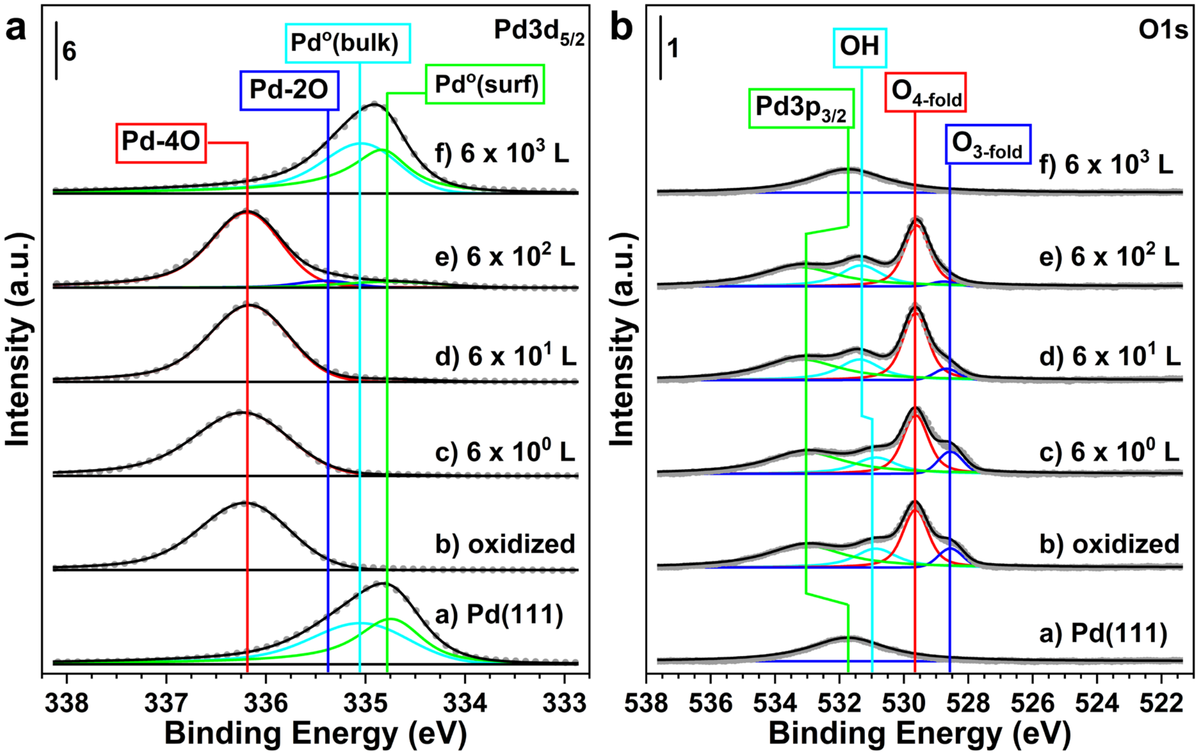

Supplementary Figure 1. Subsequent (**a**) Pd3d$_{5/2}$ and (**b**) Pd3p/O1s spectra acquired with a E_{kin} = 200 eV in UHV of (a) Pd(111), (b) after oxidation in 2 Torr of oxygen at 773 K for 5 mins, (c-f) after sequential reduction with the described hydrogen exposures at 300 K. Peak assignments: (**a**) (red) 336.2 ± 0.1 eV palladium coordinated with four oxygens, (blue) 335.4 ± 0.1 eV palladium coordinated with two oxygens, (light blue) 334.9 ± 0.1 eV bulk metallic palladium, and (green) 334.7 ± 0.1 eV surface metallic palladium. (**b**) (green) 533.2 ± 0.1 eV palladium oxide, (green) 531.8 ± 0.1 eV metallic palladium, (light blue) 531.2 – 530.9 eV hydroxyls, (red) 529.6 ± 0.1 eV 4-fold oxygen and (blue) 528.5 ± 0.1 eV 3-fold oxygen. The peak assignments are tabulated in Supplementary Table 1.

Supplementary Note 2. The surface oxide of Ag(111) is not readily reduced by hydrogen (Supplementary Figure 2). As prepared Ag(111) is characterized by one $Ag3d_{5/2}$ peak at 368.1 ± 0.1 eV, which is assigned to metallic silver (Ag^0, Supplementary Figure 2).[1,4,5] The oxidation of Ag(111) forms a silver surface oxide with an underlying metallic silver substrate as evidenced by the emergence of a $Ag3d_{5/2}$ peak at 367.6 ± 0.1 eV and enduring presence of metallic silver, respectively, along with O1s peaks at 530.15 ± 0.1 eV (disorder atomic oxygen), 528.95 ± 0.1 eV (Ag oxide at steps) and 528.15 ± 0.1 eV (p(4x4) Ag oxide) (Supplementary Figure 2).[5,6] Sequential exposures to hydrogen at pressures of 1.5×10^{-8} Torr (6×10^{-1} L), 1.1×10^{-7} Torr (6×10^{0} L), 1.1×10^{-6} Torr (6×10^{1} L), 1.0×10^{-5} Torr (6×10^{2} L), 1.0×10^{-4} Torr (6×10^{3} L), 3.0×10^{-3} Torr (6×10^{4} L), 1.3×10^{-2} Torr (6×10^{5} L) and 1.0×10^{-1} Torr (6×10^{6} L) for 1 min each at 300 K show only slight reduction (Supplementary Figure 2). The silver surface oxide is essentially unreactive with hydrogen up to 0.1 Torr.

The inability of hydrogen to reduce the silver oxide can be attributed to a high activation barrier for dissociative adsorption on both reduced and oxidized silver.[7] Indeed, theoretical calculations predict activation barriers for dissociative adsorption on O/Ag(111) and Ag(111) of 107 kJ mol^{-1} (1.11 eV) and 71 kJ mol^{-1} (0.74 eV), respectively; the activation barrier is too high to have substantial dissociation of hydrogen under the conditions examined which would readily reduce silver oxide due to the low activation barrier for the reaction of atomic hydrogen with silver oxide.

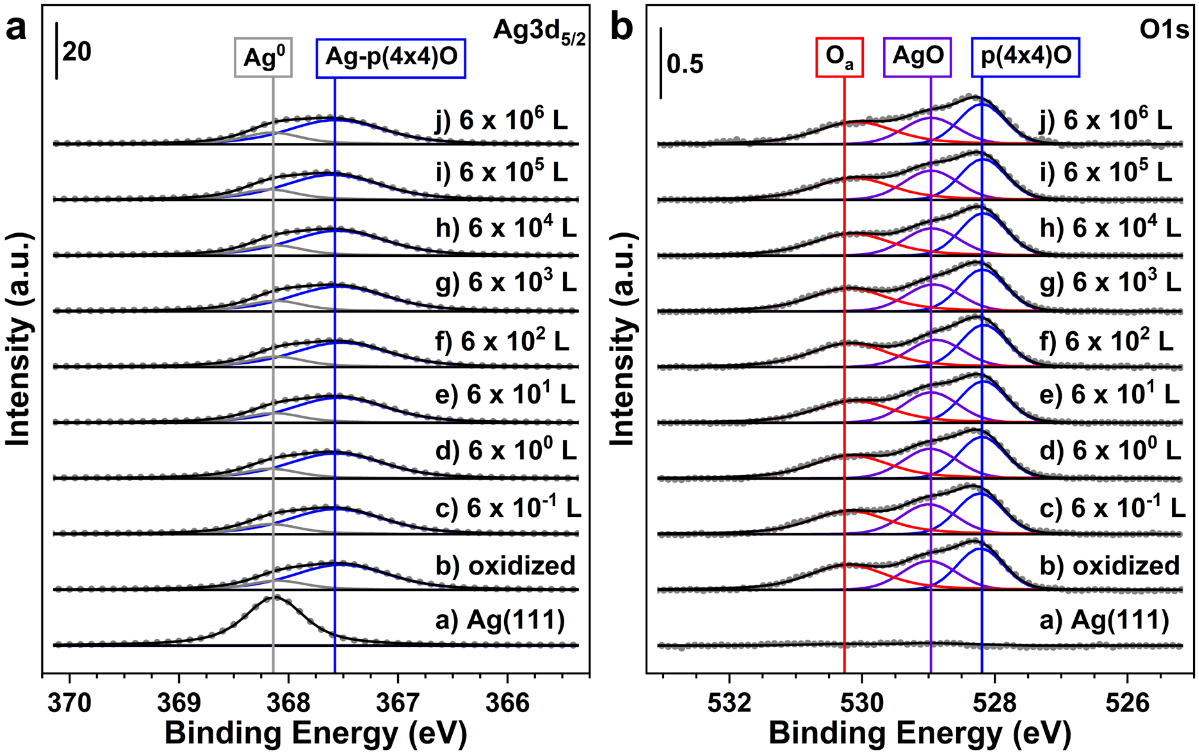

Supplementary Figure 2. Subsequent (**a**) $Ag3d_{5/2}$ and (**b**) O1s spectra acquired with a E_{kin} = 200 eV in UHV of (a) Ag(111), (b) after oxidation in 2 Torr of oxygen at 773 K for 5 mins, (c-j) after sequential reduction with the described hydrogen exposures at 300 K. Peak assignments: (**a**) (grey) 368.1 ± 0.1 eV metallic silver, and (blue) 367.6 ± 0.1 eV Ag(111)-p(4×4)O. (**b**) (red) 530.15 ± 0.1 eV disorder atomic oxygen, (purple) 528.95 ± 0.1 eV Ag oxide at steps, (blue) 528.15 ± 0.1 eV p(4x4) Ag oxide. The peak assignments are tabulated in Supplementary Table 1.

Supplementary Table 1. Binding Energies of Each Species in the Pd3d$_{5/2}$, Pd3p, Ag3d$_{5/2}$ and O1s Regions for Pd(111) and Ag(111).

Surface	Region	Species	Binding Energy (eV)
Pd(111)	Pd3d$_{5/2}$	Pd^{o}-surface	334.7 ± 0.1
		Pd^{o}-bulk	334.9 ± 0.1
		Pd-2O	335.4 ± 0.1
		Pd-4O	336.2 ± 0.1
Pd(111)	Pd3p$_{3/2}$	Pd-metallic	531.8 ± 0.1
		Pd-oxide	533.2 ± 0.1
Pd(111)	O1s	$O_{3\text{-fold}}$	528.5 ± 0.1
		$O_{4\text{-fold}}$	529.6 ± 0.1
		OH	530.9 – 531.2
Ag(111)	Ag3d$_{5/2}$	Ag-p(4×4)O	367.6 ± 0.1
		Ag^{o}	368.1 ± 0.1
Ag(111)	O1s	O_a	530.15 ± 0.1
		AgO	528.95 ± 0.1
		p(4x4)O	528.15 ± 0.1

Supplementary Note 3. The as-deposited 0.10 ML palladium on Ag(111) exhibits one $Pd3d_{5/2}$ peak at 334.8 ± 0.1 eV, which is assigned to metallic palladium (Pd^0), and two $Ag3d_{5/2}$ peaks at 368.1 ± 0.1 eV and 367.8 ± 0.1 eV, which are assigned to metallic silver (Ag^0), and silver coordinated with palladium ($Ag_{1-x}Pd_x$), respectively (Supplementary Figures 3, 4).[1,8] The oxidation of palladium on Ag(111) is characterized by the appearance of $Pd3d_{5/2}$ peaks at 336.2 ± 0.1 eV and 335.4 ± 0.1 eV, $Ag3d_{5/2}$ peak at 367.6 ± 0.1 eV and O1s peaks, which are assigned to palladium coordinated with four oxygens (Pd-4O), palladium coordinated with two oxygens (Pd-2O) and Ag(111)-p(4×4)O, respectively (Supplementary Figures 3, 4).[9] The features in the O1s region on the oxidized palladium-silver surface cannot be clearly assigned to physical features as there are several overlapping features from references on pure silver oxide[5] and palladium oxide[2] in agreement with previous assessments on oxidized $Pd_{75}Ag_{25}$(100)[10,11]. Oxidation of palladium (0.10 monolayers) on Ag(111) yielded both palladium and silver oxide and alloyed palladium-silver (Supplementary Figure 3). Metallic silver in the bulk was also detected (Supplementary Figure 3a). A depth profile of the Pd $3d_{5/2}$ photoelectron peak established that palladium oxide resides on the surface while metallic palladium is in the subsurface layers (Supplementary Figure 3b, 3c).

Subsequent hydrogen treatments to oxidized palladium on Ag(111) demonstrate that silver oxide and palladium oxide are reduced sequentially by hydrogen (Supplementary Figure 4). The oxidized surface was reduced with sequential exposures of hydrogen with a pressure of 1.2×10^{-6} Torr (6×10^1 L), 1.0×10^{-5} Torr (6×10^2 L), 1.0×10^{-4} Torr (6×10^3 L), 2.0×10^{-3} Torr (6×10^4 L), 1.5×10^{-2} Torr (6×10^5 L) and 0.11 Torr (6×10^6 L), for 1 min each at 300 K (Supplementary Figure 4). The oxidized surface did not significantly change up to a 6×10^3 L hydrogen exposure. Upon exposure to 6×10^3 L of hydrogen, the palladium oxide was only slightly reduced, as indicated by the diminishment of the Pd-4O peak along with the growth of the Pd-2O and Pd^0 peak. The slight reduction of palladium oxide is concurrent with the complete reduction of silver as evidenced by the disappearance of the Ag-p(4×4)O peak, as well as the growth of the Ag^0 and $Ag_{1-x}Pd_x$ peaks. Upon exposure to 6×10^4 L of hydrogen, the palladium oxide is further reduced. It is apparent that both the silver oxide and palladium oxide are reduced by hydrogen.

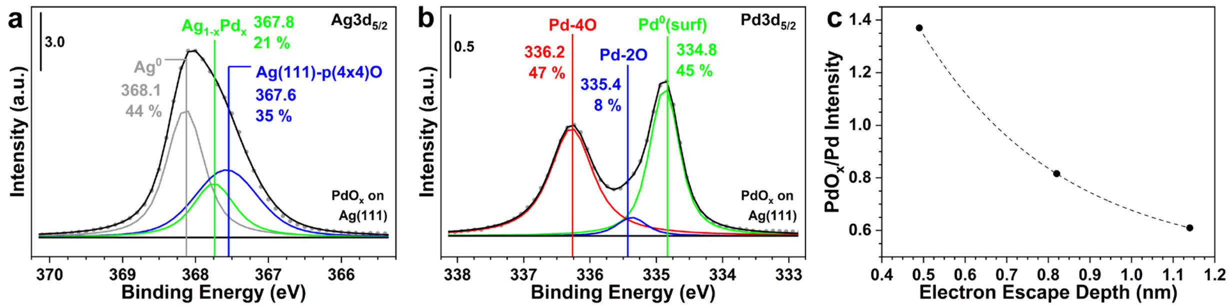

Supplementary Figure 3: Oxidation of 0.10 ML of palladium on Ag(111) forms a PdO_x surface oxide with underlying metallic palladium surrounded by a silver oxide surface. The oxidized palladium-silver surface consists of (**a**) silver oxide, alloyed palladium-silver and metallic silver as observed in Ag3d$_{5/2}$ region and (**b**) palladium oxide and metallic palladium as observed in the Pd3d$_{5/2}$ region using APXPS. (**c**) A depth profile indicates that PdO_x originates from the surface while metallic palladium is under the surface as evidenced by the decreasing PdO_x:Pd ratio with increasing electron escape depth. The dashed line is solely to guide the eye. Depth profile (kinetic energy / λ_{IMFP}(Pd)): 200 eV / 0.49 nm, 500 eV / 0.82 nm and 800 eV / 1.14 nm.[12]

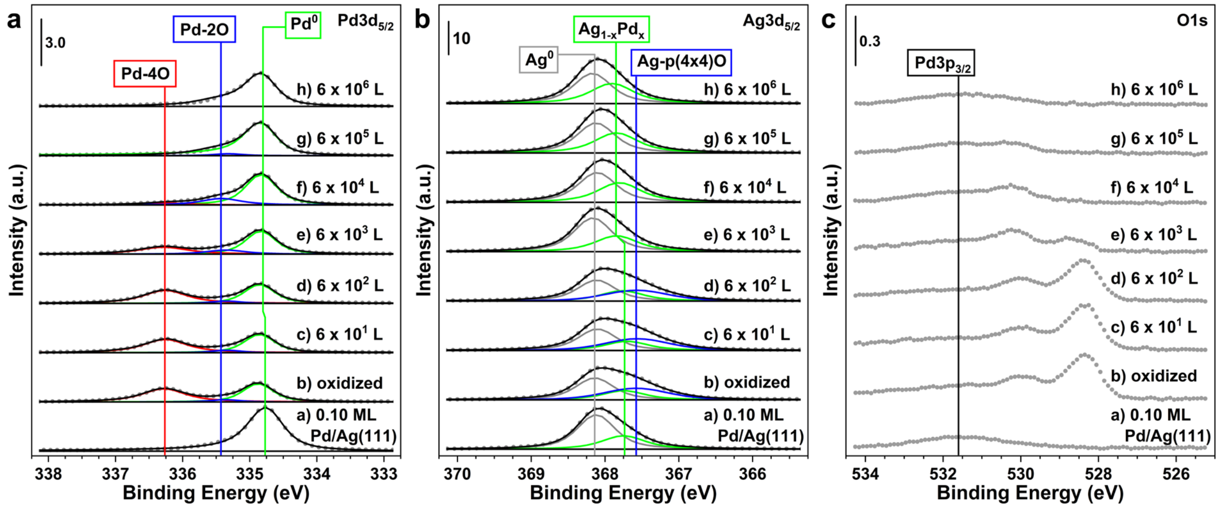

Supplementary Figure 4. Subsequent (**a**) $Pd3d_{5/2}$, (**b**) $Ag3d_{5/2}$ and (**c**) O1s spectra acquired with a E_{kin} = 200 eV in UHV of (a) 0.10 ML palladium on Ag(111) as-deposited, (b) after oxidation in 3 Torr of oxygen at 373 K for 25 mins, (c-h) after sequential reduction with the described hydrogen exposures at 300 K. Peak assignments: (**a**) (red) 336.2 ± 0.1 eV palladium coordinated with four oxygens, (blue) 335.4 ± 0.1 eV palladium coordinated with two oxygens, and (green) 334.8 ± 0.1 eV metallic palladium. (**b**) (grey) 368.1 ± 0.1 eV metallic silver, (green) 367.8 ± 0.1 eV silver coordinated with palladium and (blue) 367.6 ± 0.1 eV Ag(111)-p(4×4)O. The peak assignments are tabulated in Supplementary Table 2.

Supplementary Table 2. Binding Energies of Each Species in the $Pd3d_{5/2}$ and $Ag3d_{5/2}$ Regions for Pd/Ag(111).[a]

Region	Species	Binding Energy (eV)
$Pd3d_{5/2}$	Pd^0	334.8 ± 0.1
	Pd-2O	335.4 ± 0.1
	Pd-4O	336.2 ± 0.1
$Ag3d_{5/2}$	Ag-p(4×4)O	367.6 ± 0.1
	$Ag_{1-x}Pd_x$	367.8 ± 0.1
	Ag^0	368.1 ± 0.1

Supplementary Table 3. Percent Change in the Area of XPS Signals as referenced to Ag(111) for $Ag3d_{5/2}$ and Pd/Ag(111) for $Pd3d_{5/2}$

	**Total $Ag3d_{5/2}$**	**PdAg Alloy $Ag3d_{5/2}$**	**Total $Pd3d_{5/2}$**
**Post-oxidation of Ag(111)**	**- 9 %**		
**Post-oxidation of Pd(111)**			**- 25 %**
**Post-Pd deposition on Ag(111)**	**- 18 %**		
**Post-oxidation of Pd/Ag(111)**	**- 18 %**	**- 33 %**	**- 30 %**
**Post-reduction of oxidized Pd/Ag(111)**	**- 14%**	**+ 46 %**	**- 24%**

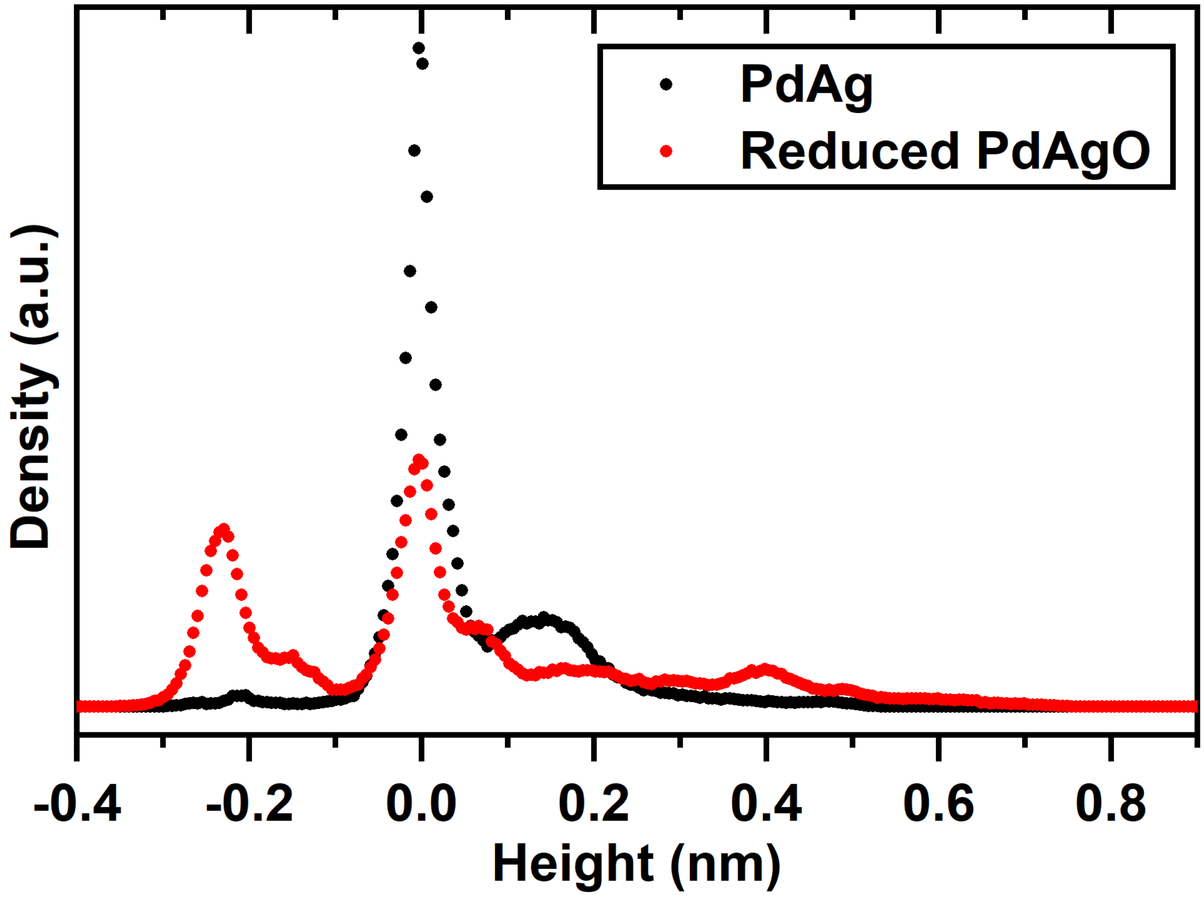

Supplementary Figure 5. The area distribution of the height of the surface with respect to the Ag(111) surface plane before deposition show that (PdAg, black) Pd/Ag(111) is characterized by a surface terrace with single layer islands while (Reduced PdAgO, red) the reduced surface is characterized by a pitted surface terrace with both single layer and double layer islands. STM details: (A) V_{sample} = 2.0 V, $I_{setpoint}$ = 0.3 nA, (B) V_{sample} = 1.5 V, $I_{setpoint}$ = 0.3 nA. The area distribution was determined from a 0.413 μm^2 region.

Supplementary Table 4. Comparative STM statistics of Pd/Ag(111) and Ag oxide surfaces

	A. 0.13 ML Pd/Ag	B. Oxidation and Reduction of A.	C. p(4x4)-AgO decomposition*	D. 0.2 ML Pd/Ag Annealed**
Island Density	0.31	0.31	-	0.38
Pit Density	0.01	0.38	0.25	0.53
Terrace Density	0.68	0.31	0.75	0.09

***Data from Klust et al.** [13]**, ** Data from van Spronsen et al.**[8]

Supplementary Note 4.

Calculation to account for silver oxide decomposition on oxidized palladium-silver reduced by hydrogen:

(Terrace density + Pit density on A.) × (Pit density on C.) = (Expected pit density on B.)

(0.69 + 0.01) × (0.25) = 0.17 pit density expected on B.

Calculation to determine pit density on Oxidized and Reduced PdAg attributed to factors other than silver oxide decomposition:

(Pit density on B.) – (Expected pit density on B.) = (Unaccounted pit density on B.)

(0.38) – (0.17) = 0.21 unaccounted pit density on B.

Calculation to approximate thermal pit density assuming linear dependence with palladium coverage:

(Pit density on D.) × (Pd coverage B.) / (Pd coverage D.) = (Expected thermal pit density on B.)

(0.53) × (0.13) / (0.20) = 0.35 expected pit density on B.

The pit density on oxidized palladium-silver reduced by hydrogen (0.38) is greater than the pit density expected from silver oxide decomposition (0.17) which suggests that the pits observed are not only due to silver oxide decomposition. This suggests that the uncounted pit density (0.21) is a result of silver intermixing into palladium islands. Directly annealing surface A. leads silver intermixing to form a thermodynamically stable structure. The amount of silver intermixing for B. is significantly less than the expected from a thermal treatment.

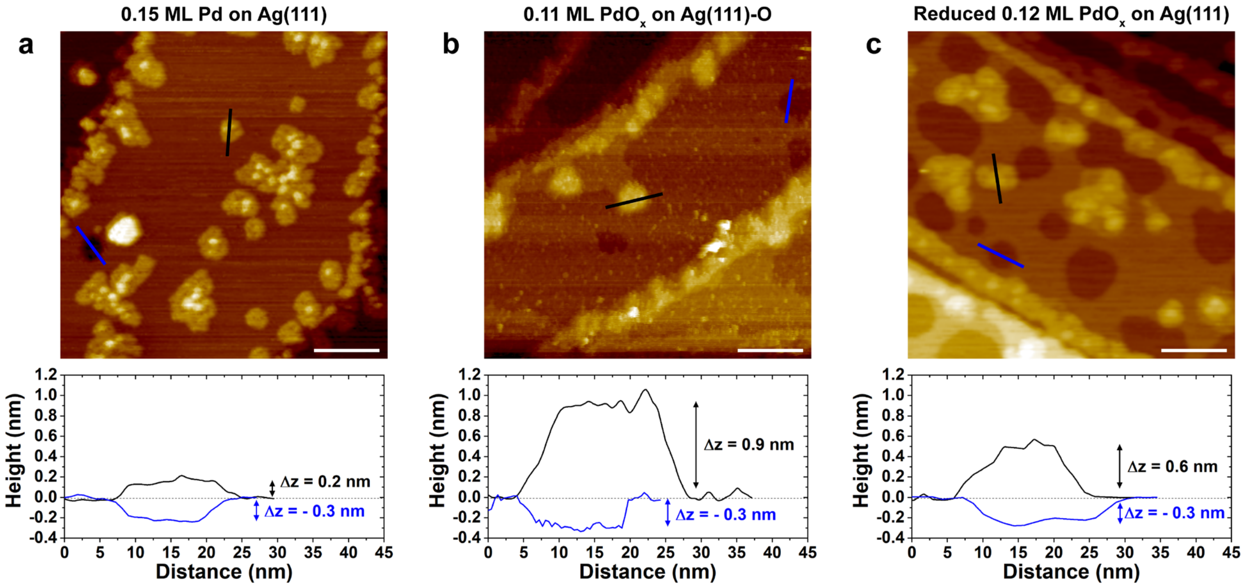

Supplementary Figure 6. A series of characteristic STM images and line scans of the structure of silver etch pits and palladium islands of (**a**) palladium on Ag(111), (**b**) after oxidation of A in 3 Torr of oxygen at 425 K and (**c**) subsequent reduction at 300 K. (**a**) The palladium islands have a uniform apparent height distribution with features of single layer palladium (0.2 nm) and second layer features (0.4 nm). (**b**) The multilayer palladium oxide islands have a uniform apparent height distribution of 0.9 nm. (**c**) The intermixed palladium-silver islands have significant height corrugation with an average apparent height of 0.6 nm. (**a-c**) Silver etch pits dominate the reduced palladium-silver surface and have an apparent height of 0.3 nm for all surfaces. Scale bars: 40 nm.

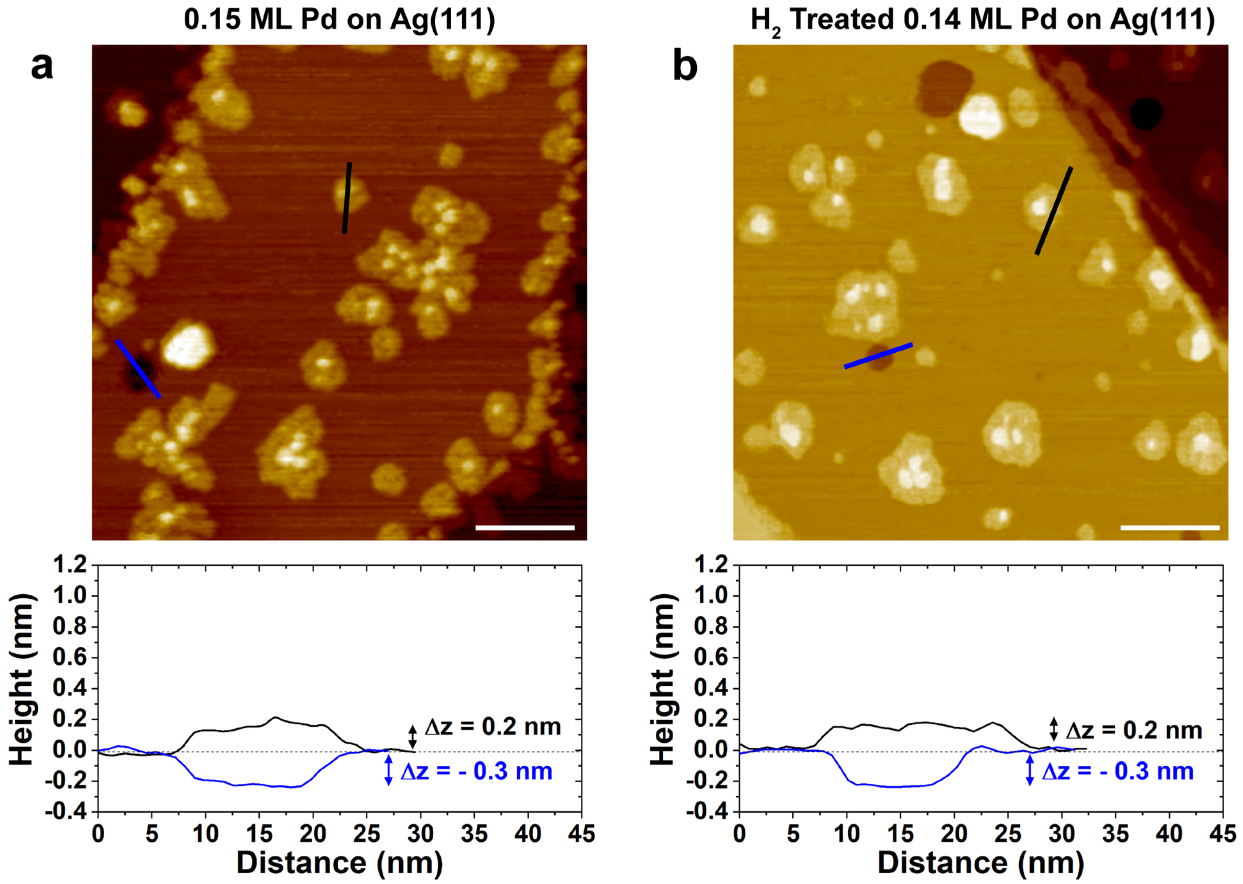

Supplementary Figure 7. (**a**) Supplementary Figure 6A is reproduced here for comparison. (**b**) A characteristic STM image of 0.14 ML of palladium on Ag(111) after exposure to 1 Torr of hydrogen at 300 K. There is only limited formation of etch pits with a height of 0.2 – 0.3 nm that is indistinguishable from that present on an as prepared palladium on Ag(111) surface. STM details: (B) V_{sample} = 1.90 V, $I_{setpoint}$ = 0.250 nA. Scale bars: 40 nm.

Supplementary References

1. Andersen, J. N. *et al.* Surface core-level shifts of some 4d-metal single-crystal surfaces: Experiments and ab initio calculations. *Phys. Rev. B* **50**, 17525–17533 (1994).
2. Ketteler, G. *et al.* In Situ Spectroscopic Study of the Oxidation and Reduction of Pd(111). *J. Am. Chem. Soc.* **127**, 18269–18273 (2005).
3. Lundgren, E. *et al.* Two-dimensional oxide on Pd(111). *Phys. Rev. Lett.* **88**, 246103 (2002).
4. Hoflund, G. B., Weaver, J. F. & Epling, W. S. Ag Foil by XPS. *Surf. Sci. Spectra* **3**, 151–156 (1994).
5. Heine, C., Eren, B., Lechner, B. A. J. & Salmeron, M. A study of the O/Ag(111) system with scanning tunneling microscopy and x-ray photoelectron spectroscopy at ambient pressures. *Surf. Sci.* **652**, 51–57 (2016).
6. Schmid, M. *et al.* Structure of Ag(111)-p(4×4)-O: No silver oxide. *Phys. Rev. Lett.* **96**, 146102 (2006).
7. Mohammad, A. B., Hwa Lim, K., Yudanov, I. V., Neyman, K. M. & Rösch, N. A computational study of H_2 dissociation on silver surfaces: The effect of oxygen in the added row structure of Ag(110). *Phys. Chem. Chem. Phys.* **9**, 1247–1254 (2007).
8. Van Spronsen, M. A. *et al.* Dynamics of Surface Alloys: Rearrangement of Pd/Ag(111) Induced by CO and O_2. *J. Phys. Chem. C* **123**, 8312–8323 (2019).
9. Kaspar, T. C., Droubay, T., Chambers, S. A. & Bagus, P. S. Spectroscopic evidence for Ag(III) in highly oxidized silver films by X-ray photoelectron spectroscopy. *J. Phys. Chem. C* **114**, 21562–21571 (2010).
10. Walle, L. E. *et al.* Surface composition of clean and oxidized $Pd_{75}Ag_{25}$(100) from photoelectron spectroscopy and density functional theory calculations. *Surf. Sci.* **606**, 1777–1782 (2012).
11. Fernandes, V. R. *et al.* Reduction behavior of oxidized Pd(100) and $Pd_{75}Ag_{25}$(100) surfaces using CO. *Surf. Sci.* **621**, 31–39 (2014).
12. Shinotsuka, H., Tanuma, S., Powell, C. J. & Penn, D. R. Calculations of electron inelastic mean free paths. X. Data for 41 elemental solids over the 50eV to 200keV range with the relativistic full Penn algorithm. *Surf. Interface Anal.* **47**, 871–888 (2015).
13. Klust, A. & Madix, R. J. Mesoscopic restructuring and mass transport of metal atoms during reduction of the Ag(111)-p(4×4)-O surface with CO. *J. Chem. Phys.* **126**, 084707 (2007).
