# Supplementary material for: Hydrogen migration at restructuring palladium–silver oxide boundaries dramatically enhances reduction rate of silver oxide
Source: Nat Commun. 2020 Apr 15;11:1844. doi: 10.1038/s41467-020-15536-x (PMC7160204; doi:10.1038/s41467-020-15536-x)
Supplement: Supplementary file 2 — Peer Review [file 41467_2020_15536_MOESM2_ESM.pdf]

Editorial Note: This manuscript has been previously reviewed at another journal that is not operating a transparent peer review scheme. This document only contains reviewer comments and rebuttal letters for versions considered at *Nature Communications.*

REVIEWERS' COMMENTS:

Reviewer #1 (Remarks to the Author):

The authors have carefully considered and addressed all my comments. I find the work to be of excellent quality and suitable for publication.

Reviewer #2 (Remarks to the Author):

As indicated in my review of the earlier submission of this manuscript, the work reported is of a very high quality and will be of considerable interest to researchers in surface science and heterogeneous catalysis. The ambient pressure XPS and STM experiments provide convincing evidence for the dramatic rate enhancement in the reduction of a surface silver oxide facilitated by transfer of atomic hydrogen which results from dissociative adsorption of H2 on PdOx islands. In addition, the authors show that PdAg intermixing occurs at the interface during the reduction process. The authors have modified the text to attempt to make a more convincing case that these reported findings are novel and not directly comparable to the effect that has been widely reported in the heterogeneous catalytic literature - namely that the rate of reduction of an oxide can be enhanced by the proximity of a material that readily dissociates H2. The revised manuscript now convincingly argues that the mechanism proposed involves transformations occurring at the interface between the palladium and silver oxides and consequently is distinguishable from the previously reported work where enhanced oxide reduction and the formation of bimetallic particles involve migration of material across an oxide carrier.

In conclusion, I recommend that the article be accepted for publication in Nature Communications.

Reviewer #3 (Remarks to the Author):

In my previous report, I recommended publication. I suggested that the authors consider a few points before publication. They provided satisfactory responses to my suggestions. I looked at the reports of the other reviewers and the author's responses. I believe that they have provided appropriate responses by making suitable revisions, or offer convincing rebuttals. Therefore, I again recommend publication.

Response to Reviewer 1

Reviewer's Comment: The authors have carefully considered and addressed all my comments. I find the work to be of excellent quality and suitable for publication.

Author Response: *We thank the reviewer for their feedback and believe that the paper now is suitable for publication in Nature.*

Response to Reviewer 2

Reviewer's Comment: As indicated in my review of the earlier submission of this manuscript, the work reported is of a very high quality and will be of considerable interest to researchers in surface science and heterogeneous catalysis. The ambient pressure XPS and STM experiments provide convincing evidence for the dramatic rate enhancement in the reduction of a surface silver oxide facilitated by transfer of atomic hydrogen which results from dissociative adsorption of H2 on PdOx islands. In addition, the authors show that PdAg intermixing occurs at the interface during the reduction process. The authors have modified the text to attempt to make a more convincing case that these reported findings are novel and not directly comparable to the effect that has been widely reported in the heterogeneous catalytic literature - namely that the rate of reduction of an oxide can be enhanced by the proximity of a material that readily dissociates H2. The revised manuscript now convincingly argues that the mechanism proposed involves transformations occurring at the interface between the palladium and silver oxides and consequently is distinguishable from the previously reported work where enhanced oxide reduction and the formation of bimetallic particles involve migration of material across an oxide carrier.

In conclusion, I recommend that the article be accepted for publication in Nature Communications.

Author Response: *We thank the reviewer for their feedback and believe that the paper now is suitable for publication in Nature.*

Response to Reviewer 3

Reviewer's Comments: In my previous report, I recommended publication. I suggested that the authors consider a few points before publication. They provided satisfactory responses to my suggestions. I looked at the reports of the other reviewers and the author's responses. I believe that they have provided appropriate responses by making suitable revisions, or offer convincing rebuttals. Therefore, I again recommend publication.

Author Response: *We thank the reviewer for their feedback and believe that the paper now is suitable for publication in Nature.*
